# Supplementary material for: Content-rich biological network constructed by mining PubMed abstracts
Source: BMC Bioinformatics. 2004 Oct 8;5:147. doi: 10.1186/1471-2105-5-147 (PMC528731; doi:10.1186/1471-2105-5-147)
Supplement: Additional File 2 — The original results of the above study (non-essential files are deleted to keep the file size under the limit set by BMC bioinformatics). [file 1471-2105-5-147-S2.bz2 › chilibotAdditionalFile2/dip05/493ID10187774E10656/html/TGFBR1_TGFB2.html]

 


 **TGFBR1** and **TGFB2** 
  
Found 21 abstracts in PubMed, retrieved 05.  
 

 What does Google say? 
 PDF only 
| .edu only 

---

**Interactive relationship** (e.g. stimulation, inhibition, etc)

**Non-interactive relationship** (e.g. studied together, co-existance, homology, etc.)

- We therefore studied the expression of UPA, UPAR, IGF2R, ALK5  [ **TGFBR1** ] , TGFBR2, TGFBR3, ENG, ALK1, TGFB1,  **TGFB2** , and TGFB3 in a series of 14 pancreatic carcinoma cell lines.  Ref: 11408925 Int J Oncol, 2001
- Although neither TGF beta2  [ **TGFB2** ]  nor TGF beta3 expression was affected by hypoxia, the TGF beta type I receptor  [ **TGFBR1** ]  was substantially upregulated within 6 h.  Ref: 11421617 J Surg Res, 2001
